# Wait a Minute or More (WAMM): a pragmatic stepped wedge cluster randomised implementation trial assessing the effect of a quality improvement programme on the proportion of infants achieving delayed cord clamping more than 60 s in infants <37 weeks’ gestation in up to 20 maternity hospitals

**DOI:** 10.1136/bmjpo-2025-003660

**Published:** 2025-12-11

**Authors:** Himanshu Popat, Kristy P Robledo, Sarah Finlayson, Melinda Cruz, Angela Cavallaro, Alpana Ghadge, Naomi Spotswood, Amy K Keir, Christoph Lehner, Sailesh Kumar, Graeme R Polglase, Dennis Bonney, Malcolm Battin, Tobias Strunk, Adrienne Gordon, Khalid Aziz, Gillian Harvey, Kei Lui, William O Tarnow-Mordi

**Affiliations:** 1 NHMRC Clinical Trial Centre, University of Sydney, Sydney, New South Wales, Australia; 2 Grace Centre for Newborn Intensive Care, The Children’s Hospital at Westmead, Westmead, New South Wales, Australia; 3 Royal Hobart Hospital, Hobart, Tasmania, Australia; 4 Burnet Institute, Melbourne, Victoria, Australia; 5 University of Melbourne, Melbourne, Victoria, Australia; 6 Women and Kids Theme, South Australian Health and Medical Institute, the Adelaide Medical School and the Robinson Research Institute, University of Adelaide, Adelaide, South Australia, Australia; 7 Royal Brisbane and Women’s Hospital, Brisbane, Queensland, Australia; 8 University of Queensland, Brisbane, Queensland, Australia; 9 Mater Research, Mater Research Institute/University of Queensland, Brisbane, Queensland, Australia; 10 Hudson Institute of Medical Research, Dept of Pediatrics, Monash University, Melbourne, Victoria, Australia; 11 Royal Darwin Hospital, Darwin, Northern Territory, Australia; 12 Auckland City Hospital, Auckland, New Zealand; 13 The Kids Research Institute Australia, The University of Western Australia, Perth, Western Australia, Australia; 14 Neonatal Intensive Care, Royal Prince Alfred Hospital, Camperdown, New South Wales, Australia; 15 Charles Perkins Centre and Reproduction and Perinatal Centre, The University of Sydney, Camperdown, New South Wales, Australia; 16 Pediatrics, University of Alberta, Edmonton, Queensland, Canada; 17 Flinders University, Adelaide, South Australia, Australia; 18 School of Women's and Children’s Health, University of New South Wales, Sydney, New South Wales, Australia; 19 Newborn Care, Royal Hospital for Women, Sydney, New South Wales, Australia

**Keywords:** Neonatology, Qualitative research

## Abstract

**Introduction:**

Delayed cord clamping (DCC) is an evidence-based intervention that reduces mortality, anaemia and disability in infants born <37 weeks’ gestation who do not require immediate resuscitation. However, it is neither reliably recorded nor routinely implemented in Australia. The Wait a Minute or More (WAMM) study aims to reduce this gap between the evidence and practice by integrating timely sharing of cord clamping data with Evidence-based Practice for Improving Quality methods to increase the proportion of preterm infants receiving DCC for 60 s or longer (DCC60).

**Methods:**

The WAMM study is a pragmatic stepped wedge cluster randomised trial (SW-CRT), informed by the Integrated-Promoting Action on Research Implementation in Health Services (i-PARIHS) framework. Up to 20 Australian maternity hospitals will participate in this pragmatic SW-CRT to evaluate if in (*Population*) infants <37 weeks’ gestation who do not need resuscitation, does (*Intervention*) the WAMM intervention (sharing of anonymised data on DCC60, together with a locally adapted quality improvement (QI) programme), compared with (*Control*) sharing of anonymised data on DCC60 alone, increase (*primary Outcome*) the proportion of infants receiving DCC60? At the end of 72 weeks, all sites will complete an 8-week period without the WAMM intervention to evaluate if implementation of DCC is sustained. Alongside the SW-CRT, an embedded process evaluation will assess the fidelity, acceptability, mechanisms of action and contextual barriers and enablers of the WAMM intervention.

**Discussion:**

Using the stepped wedged design and guided by an explicit implementation framework (i-PARIHS), WAMM will provide information on the effectiveness and transferability of a locally adapted QI method to improve DCC60. If proven effective, ultimately scaling up the WAMM intervention globally will greatly improve childhood anaemia, death, disability and long-term costs.

**Trial registration number:**

ACTRN12624000035527.

**WHAT IS ALREADY KNOWN ON THIS TOPIC**

- Delayed cord clamping (DCC), defined as clamping the umbilical cord 30–60 s or more after birth, is recommended for preterm infants not requiring immediate resuscitation. Despite strong evidence suggesting that DCC can reduce mortality and morbidity, the translation of this evidence into clinical practice remains inconsistent.

**WHAT THIS STUDY ADDS**

- Using the stepped wedged design and guided by an explicit implementation framework, this study will provide information on the effectiveness and transferability of a locally adapted quality improvement method, along with data sharing on improving the compliance to DCC.

**HOW THIS STUDY MIGHT AFFECT RESEARCH, PRACTICE OR POLICY**

- If proven effective, scaling up the study intervention globally will greatly improve childhood anaemia, death, disability and long-term costs.

## Introduction

 The World Health Organisation,^1^ the International Liaison Committee on Resuscitation^2^ and the Australian Resuscitation Council^3^ all recommend that preterm infants who do not require immediate resuscitation have their umbilical cord clamped at 60 s or more after birth, a practice known as delayed cord clamping (DCC). This guidance reflects high-quality evidence^4 5^ showing that DCC reduced death before discharge by 32% (OR 0.68, 95% CI 0.51 to 0.91)^4^ and reduced death or major disability at 2 years by 17% (relative risk 0.83, 95% CI 0.72 to 0.95).^6^ Of note, long deferral (>120 s) before clamping the cord reduced death before discharge the most (OR 0,31, 95% credibility interval 0.11 to 0.80; moderate certainty). However, individual trials of longer deferral time were relatively small and none independently demonstrated any evidence of a difference.^7^

**Figure 1:**
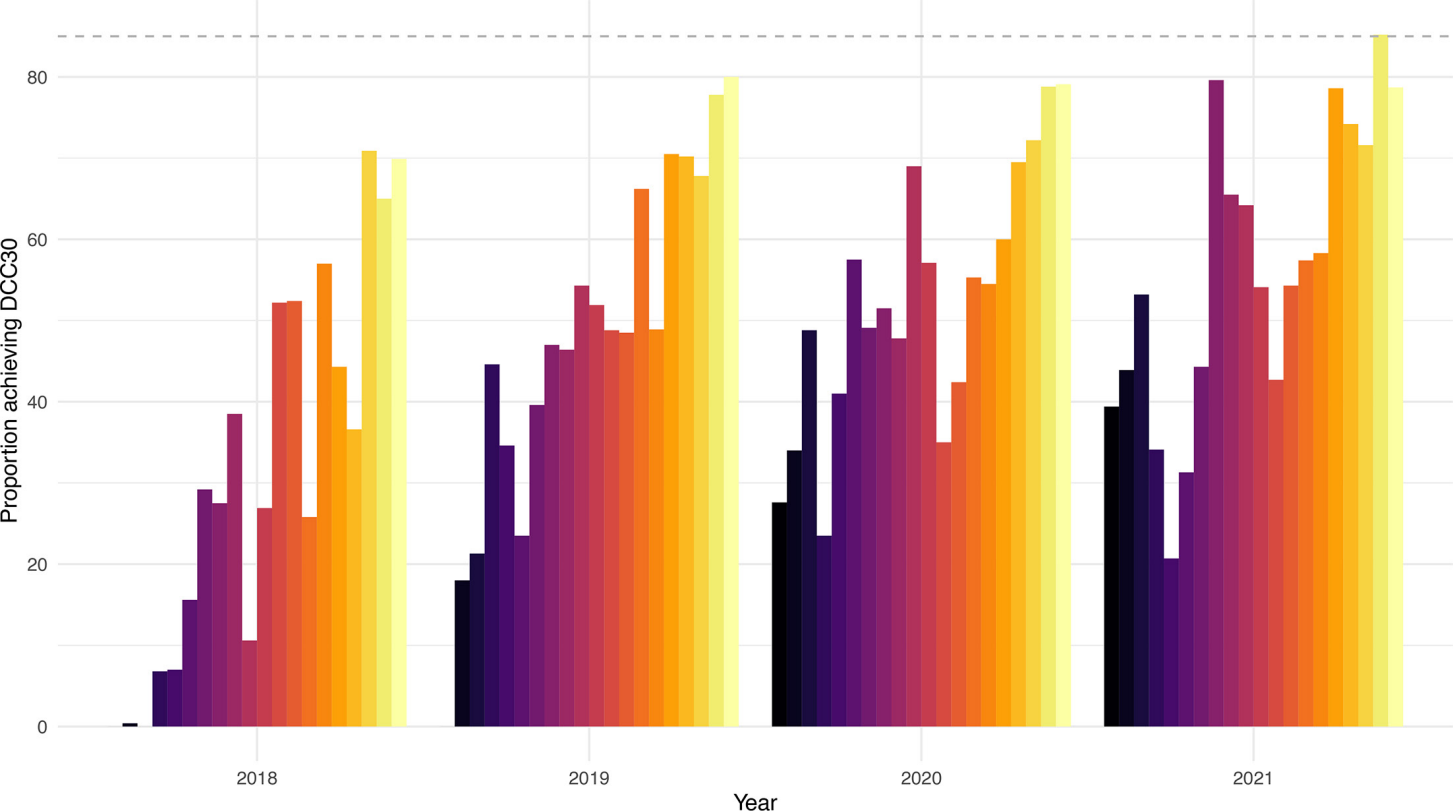
Proportion of preterm infants at each Australian and New Zealand Neonatal Network tertiary maternity hospital in Australia achieving a delay in cord clamping of at least 30 s (DCC30), over time. Each coloured bar corresponds to a hospital (produced with permission).

Although there is strong evidence suggesting that DCC can reduce mortality and morbidity, the translation of this evidence into clinical practice remains inconsistent. In Australia and New Zealand, the precise timing of cord clamping in infants born before 37 weeks’ gestation is not systematically documented. Currently, the only available data pertain to clamping performed after 30 s (yes/no) and only for infants born before 32 weeks’ gestation in all 20 tertiary maternity hospitals in Australia from the Australian and New Zealand Neonatal Network (ANZNN) (unpublished data, figure 1). Despite aiming for a compliance rate of 85% to delaying cord clamping for at least 30 s (DCC30) to accommodate immediate clamping for resuscitation in up to 15% of cases, only one of the 20 ANZNN hospitals in Australia met this benchmark between 2019 and 2021 (figure 1). Furthermore, the rates of DCC for infants born between 32 and 36 weeks are unknown and may be even lower.

Bridging the gap between evidence and clinical practice is essential, especially given the strong evidence for DCC as an effective, affordable and accessible intervention. Collaborative quality improvement (CQI), involving multidisciplinary teams and people with lived experience, has proven effective in enhancing clinical practices and outcomes. A review of 64 studies showed CQI significantly improves both care processes and patient health.^8^ The Evidence-based Practice for Improving Quality (EPIQ)^9^,^11^ method, a CQI approach based on Promoting Action on Research Implementation in Health Services (PARIHS) framework^12^ developed by the Canadian Neonatal Network, has been instrumental in reducing the combined outcome of death and illness rates in infants born before 29 weeks of gestation.^13^

The Wait a Minute or More (WAMM) study is designed as a pragmatic stepped wedge cluster randomised trial (SW-CRT) aimed at integrating DCC practices for preterm infants across up to 20 maternity hospitals in Australia. The WAMM study will use the updated i-PARIHS framework^12^ to strategise both the intervention and its implementation, and will integrate a robust process evaluation. The primary hypothesis for the WAMM study is that implementing the WAMM intervention will increase by 10% the proportion of infants achieving DCC60 (eg, from 50% to 60%, or from 60% to 70%) adjusted for trends over time.

## Methods

### Study design and setting

**Figure 2:**
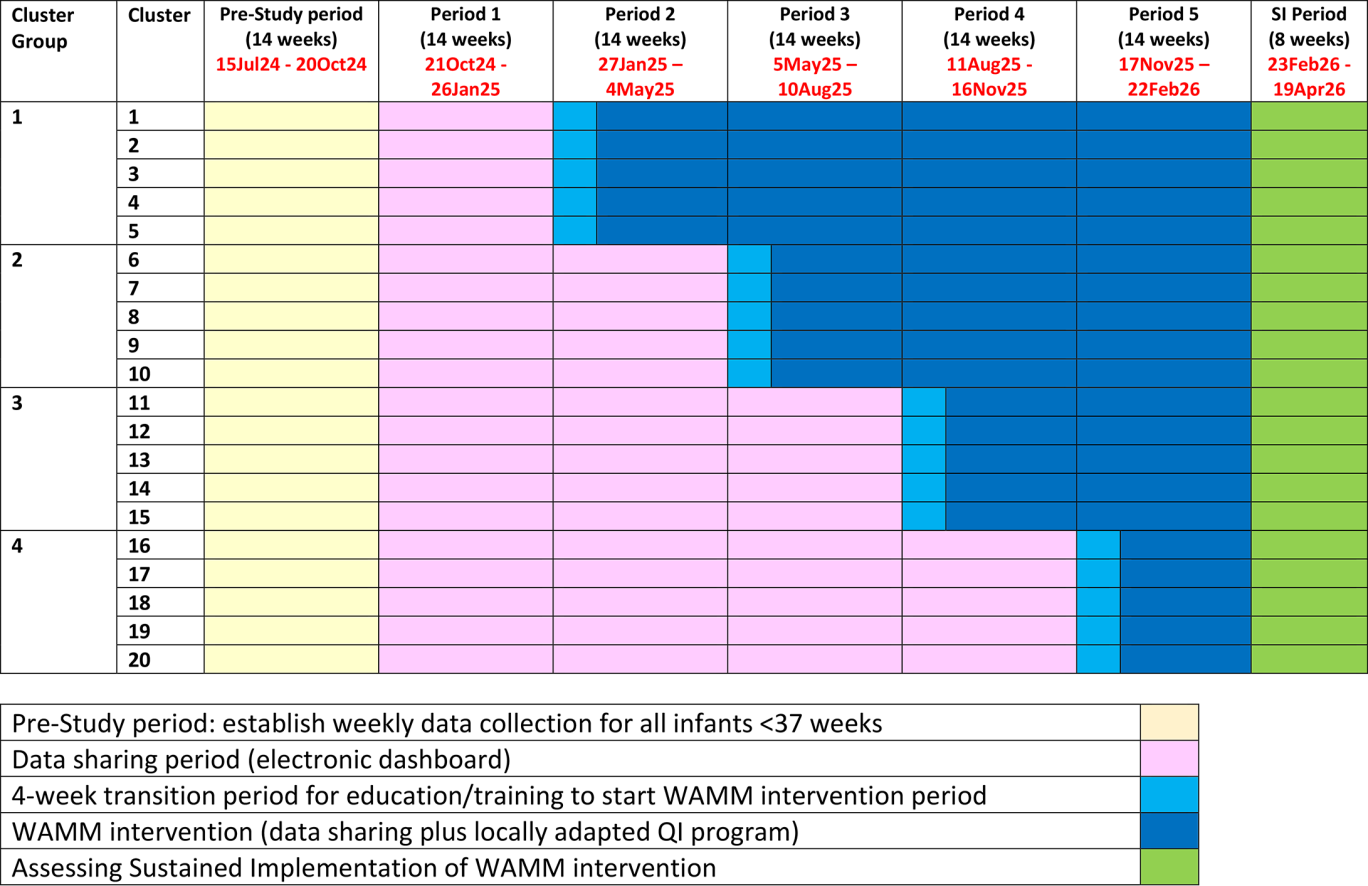
Wait a Minute or More Study: a stepped wedge cluster randomised trial.

The WAMM study will follow published guidelines for reporting SW-CRTs^14^,^17^ and Standards for Quality Improvement Reporting Excellence guidelines.^18^ Following Hargreaves *et al*’s guidance on selecting SW-CRTs,^19^ we consulted 17 neonatal directors from a representative sample of ANZNN units. 16 (94%) favoured the stepped wedge design for WAMM, in which the intervention would be introduced to 4 or 5 sites at a time (figure 2), because a parallel design would overextend resources by requiring simultaneous rollout across 9 or 10 sites. Additionally, an SW-CRT adjusted for trends over time will yield more robust evidence than a non-randomised before-and-after study design.

Up to 20 geographically representative Australian maternity hospitals will participate. This includes a mix of tertiary (delivering all infants, from 22 weeks’ gestation) and non-tertiary hospitals (delivering only infants ≥32 weeks). Although there are around 220 non-tertiary maternity units in Australia, most would not be eligible for participation due to factors like not delivering preterm infants, having low birth rate or geographic saturation. After national outreach, including a 2023 conference presentation, 18 sites agreed to participate, 8 (45%) of which were non-tertiary. Despite the significant barriers that non-tertiary unit’s experience (eg, only 13% had research staff), this level of engagement from non-tertiary units was seen as a positive outcome.

Each of the participating sites is considered a cluster (figure 2). An initial pre-study period of 14 weeks will establish the process for weekly data collection, including the number of seconds of cord clamping in all infants <37 weeks (figure 2). All sites will then enter a 14-week data-sharing period (control period, period 1). After 14 weeks, cluster groups (groups of up to five sites) will be randomised to the WAMM intervention (period 2–5) in a stepwise manner (figure 2).

### Population

#### Site eligibility criteria

Tertiary or non-tertiary Australian maternity hospitals willing and able to comply with all study requirements including intervention, timing and/or nature of required assessments for the duration of the study will be eligible. There are no exclusion criteria.

#### Infant eligibility criteria

All liveborn infants born at participating sites before 37 weeks’ gestation for whom active care is planned are eligible for inclusion in the WAMM study. Infants born before 37 weeks’ gestation and not needing immediate resuscitation will be eligible to receive DCC. Infants requiring immediate resuscitation (except at sites who have the facilities to provide resuscitation with intact umbilical cord) are ineligible to receive DCC.

### Randomisation

Sites will be randomised at the end of the pre-study period. Randomisation will be performed by minimisation using a computer-generated algorithm, stratified by hospital type (tertiary vs non-tertiary), number of infants expected in the 14-week period and average baseline number of seconds of cord clamping (NSCC) (both as determined during the pre-study period; less than the median vs greater than or equal to the median). Sites will be allocated to commence the quality improvement (QI) programme in period 2, 3, 4 or 5, with the ratio 1:1:1:1 (ie, equal chance of allocation to each period). As there is no sequential order, sites will be randomly selected for the order of randomisation. Only the WAMM statistician and trial manager will be unblinded immediately following randomisation. Allocation will be blinded to sites and other central trial operations staff until 14 weeks prior to the transition period for the allocated step for that cluster group, at which point up to 2–3 essential personnel nominated by the site will be informed to allow for staffing arrangements. All other participating staff at each site will be informed on the first day of the transition period.

### Control

#### Data entry

Funding will be provided for the duration of the study to have dedicated staff to collect and enter weekly data on DCC for all infants born <37 weeks’ gestation.

#### Data sharing

Sites will be provided with access to an electronic dashboard to view their DCC data and will also be able to see anonymised data of all other participating sites. This sharing will commence during period 1.

### Intervention

Once a site commences their WAMM intervention (box 1), further funding will be provided for a nurse and/or midwife to support the WAMM intervention for the duration of their intervention period (up until the end of period 5). A central QI facilitator and central parent facilitator will provide support to local QI teams, each of which will include one or two parents.

Box 1 **Description of Control and WAMM intervention**

**Control**

- Data entry
- Data sharing (electronic dashboard)

**WAMM intervention (data sharing plus locally adapted QI programme)**

*Components of the WAMM intervention*

- QI team building
- QI education
- Implementation of local change ideas
- Check-in monitoring
- Data sharing
- Collaborative learning

QI, quality improvement; WAMM, Wait a Minute or More.

To facilitate implementation of the WAMM intervention, the study team will use the following collaborative QI strategies.

1. *QI team building*: Each site will create a local multidisciplinary QI facilitation team (‘local QI team’), including one or more parent members when feasible, to lead the QI activities and education, and champion the culture and practice change. A team of 6–8 members is recommended.
2. *QI education*: Local QI teams will receive standardised QI education using the 6-hour EPIQ Workshop (virtual or in person),^10 11^ which involves a pragmatic approach to enable successful implementation of DCC60. EPIQ workshops will be delivered by the central QI facilitators for local QI teams in each site within the 4 week transition to the intervention.
3. *Implementation of local change ideas*: Local QI teams will use EPIQ methods through engagement and education of front-line staff to create change ideas to increase DCC60. These change ideas will be translated into the site culture using EPIQ 10 steps, including Plan–Do–Study–Act (PDSA) cycles.^10^ The aim is to implement the locally adapted QI programme within 4 weeks of transition to the intervention. The type and number of PDSA cycles may vary between the sites based on their current practice, previous and concurrent QI activities, and educational needs.
4. *Check-in monitoring meetings*: Each site will have one or more mentors from the central study team (central QI facilitators) who are experienced in EPIQ methods to help local QI teams engage front-line staff in QI and navigate the site-specific challenges. A check-in monitoring meeting of the key members of the local QI team and the central QI facilitators is recommended every 4–6 weeks to review progress.
5. *Data sharing*: Sites will be able to see their own DCC data and the anonymised DCC data of the other sites in the same intervention period. For example, with a total of 20 sites, sites 1–5 will only be able to see each other’s anonymised DCC data as they will be receiving the WAMM intervention in period 2. Sites 1–5 will not be able to see DCC data for sites 6–20 until sites 6–20 have commenced the WAMM intervention.
6. *Collaborative learning*: Sites within the intervention ecosystem will participate in quarterly virtual meetings to discuss progress and share their PDSA cycles and data to learn from each other. Preservation of anonymity, or self-identification of each site’s own DCC data, within the intervention ecosystem will be at the discretion of participating sites.

### Primary outcome

The primary outcome measure is the proportion of infants achieving a DCC60 among all participating sites during the intervention period vs the control period.

The primary outcome at study activation was ‘average number of seconds before cord clamping (NSCC)’ (V.2.0, 4 April 2024). Following the blinded Trial Executive Committee data review in May 2025, the primary outcome was updated to ‘delayed cord clamping after 60 s (DCC60)’ because the planned primary outcome (NSCC) was not normally distributed. This change has been approved by the Ethics committee (V.3.0, 17 July 2025).

### Secondary outcomes

#### Clinical effectiveness outcome

A secondary outcome measure for this study is the mean difference in the average number of seconds before cord clamping (NSCC) during the WAMM intervention period compared with the control period.

#### Balance measure

Balance measures are crucial in QI to ensure that enhancements in one area do not inadvertently cause issues in another.^20^ First recorded temperature, as a measure of inadvertent hypothermia, and death at 28 days or discharge (whichever occurs earlier) will be recorded as balancing measures.

#### Process outcomes

##### Fidelity, dose and reach

Fidelity will be evaluated in relation to the delivery of the WAMM intervention as intended. It will be measured by the following individual components:

1. Delivering the QI education programme within the 4-week transition window.
2. Establishment of local QI teams of 6–8 members in the 4-week transition window.
3. Creating and implementing one or more local change idea(s) in the 4-week transition window.
4. Ongoing check-in monitoring meetings between central QI facilitators and local QI teams on a 4–6-weekly basis after the first 4 weeks.
5. Participation in at least one quarterly virtual meeting of the study sites in the intervention ecosystem to discuss progress and share ideas.

Dose will be measured by

1. The number of check-in monitoring meetings between central QI facilitators and local QI teams.
2. The number of completed PDSA cycles for individual sites.

Reach will be measured by

1. The number of clinical staff who engage with the local QI team to design local change ideas.

These data will be extracted from WAMM check-in forms completed by local QI teams or central QI facilitators, and the communication and tracking data collected by the coordinating centre, including education attendance records (table 1).

**Table 1:** Overview of process evaluation data collection and analyses

i-PARIHS constructs	Process evaluation measures	Data source					Data analysis	
		WAMM check- in forms	Communication and activity tracking	Context mapping	Interviews	Acceptability questionnaire	Quantitative	Qualitative
Innovation and recipients	Fidelity, dose, reach	✓	✓		✓		✓	✓
	Acceptability	✓	✓		✓	✓		✓
Facilitation	Mechanisms of impact	✓			✓		✓	✓
Context	Barriers and enablers	✓	✓	✓	✓		✓	✓

i-PARIHS, Integrated-Promoting Action on Research Implementation in Health Service; WAMM, Wait a Minute or More.

##### Acceptability

Acceptability will be measured by responses to surveys, and interviews among a sample of key stakeholders. The survey will include the Acceptability of Intervention Measure, Intervention Appropriateness Measure and Feasibility of Intervention Measure.^21^

##### Mechanisms of impact

The mechanisms of impact of the intervention will be explored through semi-structured interviews using an interview guide. Interviews will be conducted with local QI team members, other hospital staff and managers to assess mechanisms relating to confidence, staff empowerment and skills and knowledge development.

##### Contextual barriers and enablers of implementation

During semi-structured interviews, hospital staff and wider stakeholders will be asked to provide specific examples of barriers to and enablers of WAMM, what worked well (or less well) in their own site and what would need to be considered for future implementation in other facilities. Supplementary information related to barriers and enablers will be extracted from the baseline context mapping, communication and activity tracking spreadsheets and check-in forms completed by local QI team members and the central QI facilitators.

### Subgroup analyses

Subgroup analyses of DCC60 will be conducted by site, gestational age (<32 weeks vs ≥32 weeks), mode of birth (caesarean vs vaginal) and multiple birth (yes vs no).

### Exploratory (hypothesis-generating) outcomes

To quantify the effect of the data sharing (electronic dashboard), the data collected in the data establishment pre-study period and the control period will be compared in a before-after model. We will explore whether any change in DCC60 is sustained in the sustained implementation period. Further exploratory analysis will be conducted to explore the heterogeneity of treatment effect over time and secular trends, as detailed in Hemming *et al.*^22^ The features of each local QI programme may also be explored (eg, inclusion of parent representative, specific change ideas) and correlated to DCC60.

### Statistics

A complete statistical analysis plan will be developed and available before any analyses are undertaken. All analyses will be performed according to the intention-to-treat principle, regardless of any delays to implementation of the QI programme. Summary information for the analyses is presented next.

#### Sample size calculation

In hospitals in the Australian Placental Transfusion Study,^23^ DCC60 was achieved in 73% of infants assigned to DCC. The primary hypothesis for WAMM is that implementing the WAMM intervention will increase DCC60 by 10% (eg, from 50% to 60%, or 60% to 70%) adjusted for secular trends (trends over time).

We assumed a (best case) scenario of 10 tertiary hospitals and 10 non-tertiary hospitals, and a birth rate of 5000 per year in the 10 tertiary hospitals and 2500 per year in the 10 non-tertiary hospitals, which yields a total of 75 000 births per 52 weeks, or around 100 000 births in 70 weeks (75 000×14 weeks * 5 periods)/52). Assuming 10% are born <37 weeks’ gestation yields approximately 10 000 preterm births, or ~100 (1000 ÷20 ÷ 5) preterm births at each site within each cluster period of 14 weeks.

Therefore, WAMM will have ~10 000 infants (~100 per cluster per time period), in four steps with up to five clusters per sequence (figure 2), yielding >99% power to detect whether the WAMM intervention results in a 10% improvement in DCC60 (from 50% to 60%) after adjusting for secular trends with a two-sided p value of 0.05, assuming an intra-cluster coefficient of 0.02, and unequal cluster sizes with a coefficient of variation of 0.8. If the number of infants is half of this (5000 total preterm births) over 18 sites (~55 preterm infants within a 14-week period), we still have >95% power to detect a 10% improvement from 50% to 60%, assuming an intra-cluster coefficient of 0.02, and coefficient of variation of 0.8. Given that the variability in a proportion is highest at 50%, any rates higher than this (eg, from 60% to 70%) will have higher power.

#### Primary estimand

The primary estimand population is infants born less than 37 weeks’ gestation that do not require immediate resuscitation within given Australian sites. Our control treatment is data collection and sharing of DCC data with staff within and across sites, compared with our WAMM intervention. Our endpoint is the proportion of infants achieving aDCC60, summarised by a cluster-average treatment effect, the risk difference (intervention minus control) with a 95% confidence interval (population-level summary). We will follow a treatment policy approach for intercurrent events (ie, intention to treat), irrespective of the commencement and duration of the WAMM intervention and any other site-specific programmes.

#### Analyses

The primary analysis will be performed at the infant level. For the primary outcome, a linear mixed model will be used, with an identity link, thereby estimating the risk difference in proportions with a 95% CI. The proportion achieving DCC60 in each treatment arm will also be provided. A clustering term will account for the clustering of infants within sites, and a time effect will be incorporated for secular trends. Interactions between the prespecified subgroups and intervention will be incorporated in the main analysis model of the primary outcome of DCC60.

A before-after model will incorporate time and cluster (site) terms to quantify the effect of introduction of data sharing, which commences in the data sharing period (period 1). The sustainability of any increase in DCC60 will be explored in a model incorporating the final period of the study (sustained implementation period) in an extension of the primary analysis model. Further extensions to the primary analysis model will be conducted to explore the treatment effect heterogeneity over time and the secular trends, as detailed in Hemming *et al*.^22^ The details of each local QI programme’s specific interventions may also be explored (eg, inclusion of parent representative) and correlated to DCC60.

Process evaluation outcomes will be summarised using means, medians or proportions as appropriate. These will be shown overall and by cluster groups (groups of up to five sites entering the intervention phase in the same period).

#### Data handling and monitoring

All data required for the monitoring and quantitative analysis of the study will be recorded within the electronic data capture system provided. Randomisation and related documentation will be outside the electronic data capture system and stored securely at the University of Sydney. Study-related documentation will be kept in a secure location and held for 15 years after the end of the study or longer, if required, according to local regulation.

Study data will be monitored in accordance with the study’s monitoring plan and using a risk-based approach. It is anticipated that central and remote monitoring will be the primary methods, with onsite monitoring undertaken as required and where indicated by ongoing risk assessment. Monitoring will be performed by the coordinating centre staff or their delegates and will include a centralised review of electronic case report forms and other study documents for protocol compliance, data accuracy and completeness. If required, onsite monitoring visits may involve review of the site file and source data verification, and/or an inspection of facilities. The coordinating centre will be given direct access to source documents and other study-related documents.

### Risk/benefit assessment

There is extremely minimal risk associated with the intervention: DCC has been established as safe and efficacious in both term and preterm infants not requiring immediate resuscitation, and QI programmes are commonplace within hospital practice. The only potential risk identified is the opportunity cost for staff time supporting the WAMM intervention; however, this will be funded as part of the study payments.

### Audit

This study may be subject to audit or inspection by representatives of the coordinating centre and study sponsor (University of Sydney), local or international regulatory bodies, the approving Human Research Ethics Committees (HREC) and local institutions, or as required by law.

### Patient and public involvement

In an online survey of parents by our partner organisation, Miracle Babies Foundation, during the design phase, 105/107 (98%) parents thought that data on how many preterm babies receive DCC60 should be routine in Australia and 106/107 (99%) supported a study of the best ways to achieve DCC60. A central parent partner representative has been involved from study conception, including on the funding application, and will provide ongoing support to parent members of local QI teams.

### Ethics

This protocol has been approved by the lead HREC, Hunter New England Human Research Ethics Committee (2023/ETH01558), and was determined to meet the requirements of the National Statement on Ethical Conduct in Human Research (2007, updated 2018). The protocol was developed by professional staff of the University of Sydney, the sponsoring organisation, and the Trial Management Committee (TMC), in accordance with the NHMRC CTC protocol template and Standard Operating Procedures.

The TMC has been convened and will operate in accordance with the TMC Charter. This Charter describes the committee’s structure, roles and responsibilities, including their remit to oversee the study planning and management on a national level. The TMC are delegated the decision-making responsibility for the study by the sponsor. The TMC will closely work in conjunction with other groups within the study governance structure to deliver the research.

### Consent

The study meets the criteria for waiver of consent for the collection of infant data, as outlined in Section 2.3.10 of the National Statement on Ethical Conduct in Human Research^24^ (2007, updated 2018) and in accordance with S95A of The Privacy Act 1988 (Cth).^25^

#### Staff and stakeholder consent to participate in interview

This study includes a limited number of semi-structured interviews with staff and key stakeholders, subject to availability of study resources as determined by the TMC. Participation in these interviews is voluntary and subject to informed consent from participating staff and stakeholders. Interview participants will have the option to review the interview transcript and complete thematic checking.

### Dissemination

We will disseminate the results to healthcare professionals, parent advisory groups, operational leaders, policymakers, government agencies and other knowledge users through presentation in national and international conferences, media sources and publication in relevant peer-reviewed journals using the Consolidated Standards of Reporting Trials extension for SW-CRT.^15^

## Discussion

This implementation trial represents a unique opportunity for up to 20 Australian maternity hospitals to experience a well-funded qualitative improvement initiative to increase the uptake of the evidence-based intervention of DCC in preterm newborn infants.

Ultimately, if it is proven effective, scaling up this multifaceted QI intervention will result in increased average uptake of DCC in all ~250 Australian maternity hospitals, and globally. This will reduce mortality, childhood anaemia, disability and their associated long-term costs.

Furthermore, the evaluation of secondary process outcomes may support wider implementation of the WAMM intervention and similar initiatives by helping others to understand better ‘what worked, what did not work and why’. The impact of the trial may thus extend beyond its aim of improving DCC through the QI education and collaborative opportunities participation affords each site.

The other unique aspect of this trial is the involvement of parents as active members within the multidisciplinary teams in each of the participating sites. This may provide a template for parents’ participation in QI initiatives in the newborn population.

## Data Availability

No data are available.
