# Supplementary material for: Wait a Minute or More (WAMM): a pragmatic stepped wedge cluster randomised implementation trial assessing the effect of a quality improvement programme on the proportion of infants achieving delayed cord clamping more than 60 s in infants <37 weeks’ gestation in up to 20 maternity hospitals
Source: BMJ Paediatr Open. 2025 Dec 11;9(1):e003660. doi: 10.1136/bmjpo-2025-003660 (PMC12699688; doi:10.1136/bmjpo-2025-003660)
Supplement: Supplementary file 1 [file bmjpo-2025-003660.reviewer_comments.pdf]

PEER REVIEW HISTORY

BMJ Paediatrics Open publishes all reviews undertaken for accepted manuscripts. Reviewers are asked to complete a checklist review form and are provided with free text boxes to elaborate on their assessment. These free text comments are reproduced below.

ARTICLE DETAILS

**TITLE (PROVISIONAL)**	Wait a Minute or More (WAMM): A pragmatic stepped wedge cluster randomised implementation trial assessing the effect of a quality improvement program on the proportion of infants achieving delayed cord clamping more than 60 seconds in infants <37 weeks' gestation in up to 20 maternity hospitals.
**AUTHORS**	Popat, Himanshu; Robledo, Kristy P; Finlayson, Sarah; Cruz, Melinda; Cavallaro, Angela; Ghadge, Alpana; Spotswood, Naomi; Keir, Amy; Lehner, Christoph; Kumar, Sailesh; Polglase, Graeme; Bonney, Dennis; Battin, Malcolm; Strunk, Tobias; Gordon, Adrienne; aziz, khalid; Harvey, Gillian; Lui, Kei; Tarnow-Mordi, William O.

VERSION 1 - REVIEW

**REVIEWER NAME**	Anup Katheria
**REVIEWER AFFILIATION**	Sharp Healthcare
**REVIEWER CONFLICT OF INTEREST**	N/A
**AI DISCLOSURE**	No
**DATE REVIEW RETURNED**	24-May-2025

**GENERAL COMMENTS**	I'm struggling a lot with the actual intervention on how to improve DCC rates.<br><br>There will be a central QI facilitator and a central parent facilator providing support to local QI teams…<br>What does that mean? How do they provide support? Do they attend deliveries and reassure the OB to wait the full minute.<br>Then three are 6-8 team members of the QI team? What do they do? What QI activities and education are they doing? Are they doing morning huddles? Discussing each delivery before it happens.<br>More detail about the QI education would be helpful. What will the 6-hour EPIQ workshop look like? It say it will be hands on? Do participants "practice" holding a baby with an artificial cord for 60 seconds?<br>This protocol paper is lacking a lot of depth and detail before it can be finalized.

**REVIEWER NAME**	Ju Lee Oei
**REVIEWER AFFILIATION**	Mater Research Institute The University of Queensland
**REVIEWER CONFLICT OF INTEREST**	N/A
**AI DISCLOSURE**	No
**DATE REVIEW RETURNED**	31-May-2025

**GENERAL COMMENTS**	Review Popat WAMM protocol

	This manuscript describes the protocol of a quality improvement project aimed to increase the time before cord clamping in preterm infants using a pragmatic stepped wedged cluster randomised trial. The study will include 20 Australian maternity hospitals and span 72 weeks. The main rationale for this study is that time to cord clamping in infants born before 37 weeks gestation is not systematically document in Australia and New Zealand<br>I found the protocol hard to read – there are many abbreviations. The main issue I have is that there is no background data even though that is step 1 in this study – to obtain background data. The rationale from the study is based on unpublished ANZNN data.<br>How do the authors explain this rationale?<br>Furthermore, what is the implication of change in practice on the largest population, 32-36 week infants in a developed country? This population has a low mortality rate and although at higher risk of neurodevelopmental and other health issues, cannot be compared to the population from the APTS study (<30 weeks gestation)<br>My other queries:<br>1. Line 16 Page 8 – infants born before 37 weeks GESTATION<br>2. What is the reference for the figures stated in line 25 (only 1 in 20 hospitals met benchmark)?<br>3. How was fig 1 developed if data were unpublished?<br>4. The 17 neonatal directors who were consulted were from tertiary centres. Were the opinions of non-tertiary centres sought?<br>5. Line 10: page 10 – who determines infant eligibility at each site? How is need for “resuscitation” determined?<br>6. Page 14 line 28 – please explain tertiary and non tertiary hospitals<br>7. Is there a minimum number of births for inclusion in the study?<br>8. What is the likelihood that sites will change practice during the data sharing period once it is known that they will take part in the study?<br>9. How are multiple births treated?<br>10. How are different gestations treated?<br>11. How is expertise on the floor treated? Would staff from a tertiary centre be more able to increase DCC than non-tertiary staff?<br>12. Do QI initiatives include all staff including obstetricians and allied health?<br>13. How will bias for hospitals be addressed? I.e. would there be distribution of hospitals that have lower rates/duration of cord clamping with those already practising at high rates and longer duration?<br>14. How was the sample size of 20 reached? How are site drop outs handled?

VERSION 1 – AUTHOR RESPONSE

Reviewer: 1

Anup Katheria, Sharp Healthcare

Comments to the Author

I’m struggling a lot with the actual intervention on how to improve DCC rates.

There will be a central QI facilitator and a central parent facilitator providing support to local QI teams…

What does that mean? How do they provide support? Do they attend deliveries and reassure the OB to wait the full minute.

Then three are 6-8 team members of the QI team? What do they do? What QI activities and education are they doing? Are they doing morning huddles? Discussing each delivery before it happens.

WAMM study is a multicentre (up to 20 sites) implementation trial. There are tertiary sites (delivering babies from 23 weeks gestation) and non-tertiary sites (delivering babies from 32 weeks gestation) participating in the trial.

The central QI facilitator is an expert in EPIQ methodology, and the central parent partner is an expert in consumer engagement. A core component of EPIQ methodology is that site-based barriers, facilitators, and solutions are contextual, so decisions on educational, personnel, or material interventions will differ by setting.

The central facilitator will support the local site QI teams, educating and advising the team on QI methodology and implementation, and guiding the local QI site teams to identify enablers and barriers, and suggest strategies, as required. The central parent partner will provide peer-support to the local parent member who is part of the site QI team. The local QI team will be responsible for implementing the changes. The central team will have regular check-in meetings with the local site QI teams to provide feedback and advice on the QI implementation.

The individual change ideas will be different for each site, based on the local enablers and barriers identified by that local QI team.

For example, some change ideas could be (1) explicitly specifying the indication and contraindications for delayed cord clamping in their guideline, (2) educating the staff around the evidence of delayed cord clamping, (3) senior paediatric or neonatal attendance at the delivery, (4) local QI teams attending the morning huddle and the local QI coordinator discussing each delivery as it happens (as suggested by the reviewer).

As described in the main document, local QI teams will use EPIQ methods through engagement and education of front-line staff to create change ideas to increase average number of seconds before cord clamping (NSCC). These change ideas will be translated into Plan-Do-Study-Act (PDSA) cycles using EPIQ's 10 steps. The type and number of PDSA cycles may vary between the sites based on their current practice, previous and concurrent QI activities, and educational needs.

At the end of the study, we will publish the change ideas that were adopted by different sites.

More detail about the QI education would be helpful. What will the 6-hour EPIQ workshop look like? It say it will be hands on? Do participants "practice" holding a baby with an artificial cord for 60 seconds?

This protocol paper is lacking a lot of depth and detail before it can be finalized.

The protocol is for up to 20 sites in Australia and the EPIQ workshop is a well-established methodology to implement evidence-based interventions. As described in sections a-f under the heading "Intervention" these steps include forming a multidisciplinary team, receiving the standardised education, formulate and implement change ideas, attend check-in meetings, review shared data and participate in collaborative learning.

We would like to emphasise that a core component of EPIQ methodology is that site-based barriers, facilitators, and solutions are contextual, so decisions on educational, personnel, or material interventions will differ by setting.

We believe the intervention is described in sufficient details (582 words) including a table in the methods section. We have removed the word “hands-on” and replaced it with “pragmatic” as we agree it may be interpreted incorrectly.

Reviewer: 2

Prof. Ju Lee Oei, Mater Research Ltd

Comments to the Author

Review Popat WAMM protocol

This manuscript describes the protocol of a quality improvement project aimed to increase the time before cord clamping in preterm infants using a pragmatic stepped wedged cluster randomised trial. The study will include 20 Australian maternity hospitals and span 72 weeks. The main rationale for this study is that time to cord clamping in infants born before 37 weeks gestation is not systematically document in Australia and New Zealand

I found the protocol hard to read – there are many abbreviations.

We have followed the convention of describing the abbreviation in full at its first use. We would be happy to expand some, or all the abbreviations as directed by the editorial committee.

The main issue I have is that there is no background data even though that is step 1 in this study – to obtain background data. The rationale from the study is based on unpublished ANZNN data.

How do the authors explain this rationale?

We were surprised to discover that in a high-income country like Australia, there is no data collected and published on delayed cord clamping (defined as clamping more than 60 seconds). Since this is such a low cost and effective intervention, we requested the data from the ANZNN registry (at a national level) and were surprised to discover that they only collect data for babies <32 weeks gestation, and that data is DCC30 (clamping more than 30 seconds, yes/no). This is the only data we have at the national level. We mention the source of the data and acknowledge that this is unpublished data. We do strongly believe that ANZNN is a reputed registry and follows a rigorous process to ensure the accuracy of its data. We have obtained necessary permission from ANZNN to include this information.

Furthermore, what is the implication of change in practice on the largest population, 32-36 week infants in a developed country? This population has a low mortality rate and although at higher risk of neurodevelopmental and other health issues, cannot be compared to the population from the APTS study (<30 weeks gestation)

We agree that APTS study evidence cannot be extrapolated to infants born between 32-36 weeks gestation. Hence, we cite the WHO and ILCOR references that recommend that preterm (< 37 weeks) infants who do not require immediate resuscitation have their umbilical cord clamped at 60 seconds or more after birth, a practice known as delayed cord clamping (DCC).

My other queries:

1. Line 16 Page 8 – infants born before 37 weeks GESTATION

Added.

2. What is the reference for the figures stated in line 25 (only 1 in 20 hospitals met benchmark)?

The reference is figure 1. This is now again specified after the sentence

3. How was fig 1 developed if data were unpublished?

The data was kindly provided by ANZNN registry and was unpublished at the time of protocol development. It will be considered published after this paper. We have obtained necessary permission from ANZNN to include this information.

4. The 17 neonatal directors who were consulted were from tertiary centres. Were the opinions of non-tertiary centres sought?

All 17 neonatal directors were from tertiary centres. No non-tertiary centres responded

5. Line 10: page 10 – who determines infant eligibility at each site? How is need for “resuscitation” determined?

All preterm infants are eligible for WAMM. The clinicians caring for the infants determine the need for resuscitation. The EPIQ workshop and the check in meetings encourage the clinicians to clarify and discuss various issues including the determination of the need for resuscitation. We anticipate that perception of the need for resuscitation may vary between sites. Check-in meetings and quarterly meetings as part of a collaborative ecosystem (as part of the WAMM intervention) will encourage discussion on this issue.

6. Page 14 line 28 – please explain tertiary and non tertiary hospitals

We define tertiary and non-tertiary hospitals in the previous paragraph. This includes a mix of tertiary (delivering all infants, from 23 weeks gestation) and non-tertiary hospitals (delivering only infants ≥32 weeks).

7. Is there a minimum number of births for inclusion in the study?

There is no minimum number of births for inclusion in the study.

8. What is the likelihood that sites will change practice during the data sharing period once it is known that they will take part in the study?

The stepped wedge design will allow us to investigate the effect of time and help answer this question. Specifically, we have prespecified a comparison of the data collection period to the first data sharing period. It is difficult to comment on the likelihood that sites will change practice during the data sharing period once it is known that they will take part in the study.

9. How are multiple births treated?

We do not recommend that multiple births be treated any different, with regards to the suitability for delayed cord clamping. Subgroup analyses of average NSCC will be conducted by site, gestational age (<32 weeks versus ≥32 weeks), mode of birth (caesarean versus vaginal) and multiple birth (yes versus no).

10. How are different gestations treated?

We do not recommend any special recommendation in regard to gestation strata within 23-37 weeks gestation.

Subgroup analyses of average NSCC will be conducted by site, gestational age (<32 weeks

versus ≥32 weeks), mode of birth (caesarean versus vaginal) and multiple birth (yes versus

no).

11. How is expertise on the floor treated? Would staff from a tertiary centre be more able to increase DCC than non-tertiary staff?

Expertise on the floor will be considered in each site’s specific barriers, facilitators and solutions. Staff expertise may or may not play a role in the success of each site’s efforts to increase rates of DCC: we will be able to evaluate this at the conclusion of the study.

12. Do QI initiatives include all staff including obstetricians and allied health?

Yes, QI initiatives may include all staff including obstetrics, midwifery, paediatric, neonatal medical and nursing, depending on the particular change ideas identified by the local site team. We encourage parents to be part of the local QI team. We have not specified allied health staff.

13. How will bias for hospitals be addressed? I.e. would there be distribution of hospitals that have lower rates/duration of cord clamping with those already practising at high rates and longer duration?

Firstly, the timing of rollout of the intervention at each site is randomised. Randomisation will be performed by minimisation, using a computer-generated algorithm, stratified by hospital type (tertiary versus non-tertiary), number of infants expected in the 14-week period, and average baseline NSCC

(both as determined during the Pre-study period; less than the median versus greater than or equal to the median). Secondly, due to the step wedge design, all sites start in the control phase, enabling within-hospital comparisons. Lastly, the model applied to the data accounts for site, and temporal trends over time, allowing the isolation of the intervention effect.

14. How was the sample size of 20 reached? How are site dropouts handled?

The choice of the number of clusters within a stepped wedge trial is based on a combination of statistical, logistical and practical considerations. Firstly, with more clusters, we have greater statistical power for the trial, increased precision in estimates, lower risk of bias and higher generalisability. This is balanced by the availability of eligible sites, and the practical number of sites that we could fit into the trial timeframe given the stepped wedge design. We conducted extensive feasibility surveys prior to the trial, to identify availability of eligible sites for inclusion in the trial.

While we aimed for 20 sites, we have had 18 sites formally start the trial and commence the first period. This still gives us high power to detect a treatment effect. All 18 sites have committed to conducting the trial for the whole design period.

VERSION 2 - REVIEW

**REVIEWER NAME**	Ju Lee Oei
**REVIEWER AFFILIATION**	Mater Research Institute The University of Queensland
**REVIEWER CONFLICT OF INTEREST**	N/A
**AI DISCLOSURE**	No
**DATE REVIEW RETURNED**	13-Aug-2025

**GENERAL COMMENTS**	I thank the authors for answering my queries. I understand that my of my questions stem from the unknown (i.e. lack of data, lack of response from the non-tertiary centre) and that much of the premise of this proposal was based on assumption. I still have considerable difficulty with the primary outcome - what is the reason for the change, what is the reason FOR the primary outcome and why the lack of response from the non-tertiary centres (which deliver many 32-36 week infants) was not chased up. As it stands, the revised version of the protocol is confusing and hard to appreciate. I will defer to the editor as to whether a major revision or a rejection is warranted.

VERSION 2 – AUTHOR RESPONSE

Reviewer comment: As it stands, the revised version of the protocol is confusing and hard to appreciate. I will defer to the editor as to whether a major revision or a rejection is warranted.

We appreciate the reviewer’s candid feedback and acknowledge that this quality improvement and implementation trial is not a typical trial design, nor a typical trial intervention. We have taken this opportunity to minimise the use of abbreviations, simplify terminology, clarify participation of tertiary and non-tertiary sites, and justify the change in primary outcome. The changes are reflected in the tracked version of the manuscript. The terminology we have employed is specific to the stepped wedge trial design, and we have included a Figure 2 with simplified legend to clarify this design. We describe the intervention in detail including table 1 and the points a-f under intervention section.
